# Albuminuria in Pregnancy
*Paper opening a discussion at at meeting of the Bath, Bristol and Somerset Branch of the British Medical Association held on 25th May, 1932.


**Published:** 1932

**Authors:** J. A. Nixon

**Affiliations:** Professor of Medicine, University of Bristol; Physician, Royal Infirmary, Bristol


### ALBUMINURIA IN PREGNANCY.*

BY

J. A. Nixon, C.M.G., M.D., F.R.C.P.,

*Professor of Medicine, University of Bristol;*
*Physician, Royal Infirmary, Bristol.*

---

Dr. Statham's account of albuminuria in pregnancy from the obstetrician's point of view is admirable and clearly intelligible, and he has handled the problems of treatment in such a practical fashion as to leave very little for the physician to add on this aspect. In fact, the position is this: that in all cases where a pregnant patient is known to have a pre-existing nephritis Dr. Statham leaves it to a physician to decide whether the pregnancy should proceed or be terminated. Furthermore, in a certain number of cases the obstetrician, feeling uncertain as to whether the case is one of pre-eclamptic albuminuria or nephritis, the pathologist and the physician are invited to help in the decision.

Dr. Statham speaks of a group of cases in which albuminuria has no pathological significance, but he is doubtful whether there really is any such group. As he wisely observes, these cases should be carefully investigated before deciding that the albuminuria is of no significance. He continues to say that the condition calls for careful observation during pregnancy, though it is seldom associated with any real pathological symptoms. There are two important

* Paper opening a discussion at a meeting of the Bath, Bristol and Somerset Branch of the British Medical Association held on 25th May, 1932.

reservations in this last remark : one is implied in the "careful investigation," and we may have noticed that careful investigation brings down the proportion of cases in which the albuminuria was of no pathological significance to a very low figure. The second reservation was that they seldom produce any real pathological symptoms. A word of warning is necessary here : I think that though no pathological symptoms may be associated with a mild albuminuria it is impossible to feel quite sure that no damage will result therefrom in after years.

Dr. Statham says that it is interesting to note that there are definite but slight changes in the urinary tract during every normal pregnancy. The slight changes in the renal parenchyma have been given the name of "pregnancy kidney."

Many authors speak of the strain of pregnancy upon the kidneys, and Dr. Statham, in conversation with me, has asked, has almost challenged me, to define what is meant by "the strain of pregnancy." In normal pregnancy there are definite changes in the maternal metabolism ; there must be, for example, some retention of nitrogen for the development of the fœtus. There is a marked gain in the mother's weight due to growth of the uterus, increase in the size of the breasts and, in many instances, increase in the mother's weight from storage of fat. The maternal organs have also to detoxicate and excrete certain metabolic products arising from the fœtus ; it is true that these are physiological changes and the extra burden increases by very slow degrees, but nobody would dispute, least of all no woman who has had experience of pregnancy, that the extra burden upon the entire system is distinctly perceptible before the pregnancy has come to full term. The extra burden falls chiefly, no matter how or why, upon the liver and kidneys. It has been suggested by Weichardt[1] that the damage is done, at least to the liver, by the absorption into the general circulation of toxins of enzyme nature derived from the action of certain cells of the ovum, the syncytium.

It has been claimed there is a specific change in the liver during pregnancy, evidenced by a mild degree of fatty degeneration of the cells about the centre of the lobules interfering with the glycogenic function. Von Leyden[2] demonstrated a similar condition of the kidneys; the lesion consists of a slight degree of fatty degeneration of the tubules, not infrequently accompanied by albuminuria. If these observations are not erroneous, I think it must be allowed that the effects of the abnormal strain in pregnancy have been proved to exist. The causes are more difficult to define, but Gibberd has summed the matter up when he says that "pregnancy is the most delicate test of renal efficiency." I do not think that he means that the test is not a very heavy one.

Whitridge Williams[3] speaks of a "low reserve kidney," by which he means that some women have slightly defective kidneys which are efficient under ordinary conditions but break down under the strain of pregnancy. I have often wondered if this defect is, in fact, only a slight one. You all remember how much of two healthy kidneys has to be removed before the kidneys show any impairment of function. Some observers go so far as to say that three-quarters of the total kidney substance must be destroyed before, for instance, nitrogen retention occurs. It is safer, I believe, to assume that the differences between what Whitridge Williams calls "ordinary conditions" and the conditions of pregnancy are so great that the strain of pregnancy must indeed be immense. Alternatively it must be held that the kidneys do not show themselves to be even slightly defective until they are on the point of breaking down under a very moderate strain. The recovery of these women and their continuance in good health with kidneys that are functionally unimpaired suggests that it is the strain of pregnancy that is great rather than the renal defect that develops under a slight strain.

One of the most difficult problems is to decide whether, when an albuminuria is discovered in pregnancy, it is safe to allow the pregnancy to proceed. Dr. Statham speaks of cases of previous nephritis associated with pregnancy, and I think that he has dealt with this group, but it is much more difficult when the pregnant patient is not known to have a pre-existing nephritis. Fortunately, it does not matter in the least so far as the conduct of the existing pregnancy is concerned.

Whitridge Williams's rules are a safe guide to procedure: "When symptoms of renal insufficiency appear before the child is viable they are almost invariably due to nephritic toxæmia; the earlier they appear the more certainly this is true. After the seventh month it is usually impossible to distinguish by ordinary clinical means between nephritic and pre-eclamptic toxæmia."

Many toxæmic patients diagnosed as pre-eclamptic are really nephritic, especially those women who habitually give birth to premature infants. They remain perfectly well until at a certain period in the pregnancy, very often about the same month, œdema and albuminuria suddenly develop. These cases are nearly always nephritic. For it should be noted that hepatic, pre-eclamptic or eclamptic toxæmia are commonest in first pregnancies, and that the prognosis for subsequent pregnancies is good, or, as Whitridge Williams says, "one attack of pre-eclampsia or eclampsia confers a relative immunity." Therefore toxæmia in repeated pregnancies may be taken as evidence of chronic nephritis.

Here a digression may be permitted to utter a warning against confusing chronic nephritis with syphilis as a cause of repeated miscarriage. Chronic nephritis ranks second to syphilis amongst the causes of intra-uterine death, but whereas fœtal deaths due to nephritis will occur as a rule towards the same time in successive pregnancies, the virulence of the syphilitic poison on the embryo will be found to diminish in successive pregnancies in accordance with Diday's [4] law of habitual decrease, which I give in his own words: "On examination of the cases in which syphilitic parents have had many children in succession . . . the disease affected the earliest most severely and became ameliorated afterwards in proportion as its victims were multiplied. In the first pregnancy abortion occurs in the fifth month, in the second at a later period. The result of the third is a child at full term but weakly and not viable, the fourth child is born with a constitution more capable of resistance."

When albuminuria occurs in the first pregnancy, it is not of much moment to try and distinguish between nephritis and pre-eclampsia; the treatment is identical and Dr. Statham has described it, and he concludes by saying that in most cases there is an immediate and rapid improvement, in which case the pregnancy is allowed to continue. In a certain number of cases he says the symptoms persist or even get worse in spite of the treatment, and it becomes necessary to terminate the pregnancy. If the albuminuria continues without clinical symptoms the pregnancy is allowed to proceed. In cases of albuminuria with headache, vomiting, œdema, etc., the rules for terminating the pregnancy are perhaps easier to define than the rules for differentiating nephritic from pre-eclamptic toxæmia. Marked nitrogen retention occurs in cases of pregnancy with chronic nephritis, and may or may not occur before the onset of eclampsia. It is a safe rule to terminate the pregnancy as soon as the blood-urea and non-protein nitrogen content rise above 40 mgms. per 100 c.c.

Dr. Statham mentions two laboratory tests which have proved of great value to him in clinching the diagnosis of pre-eclampsia, namely the diastase reaction which he tells us is always very high in eclampsia and pre-eclampsia, but reduced in most cases of chronic nephritis. I don't believe it matters a bit to know the diastase content in a nephritic case; the decision to terminate the pregnancy will not depend upon the diastase concentration. I think Stafford and Addis [5] are right in saying the determinations of the diastase content of the blood or urine of pregnancy with Bright's disease have no clinical value. By this I do not mean to question the clinical importance of the very high concentration of urinary diastase, which Dr. Statham says gives such valuable warning in pre-eclamptic states.

His second test was the ammonia-nitrogen : urea ratio, which always rises when the chief lesion is in the liver. But it should be emphasized that no obstetrician will refrain from terminating a pregnancy because the diastase concentration and the ammonia-nitrogen : urea ratio are within normal limits, provided that there is evidence of high nitrogen retention; and as McLean and de Wesselow[6] pointed out in 1918, no clinical information is obtained by the estimation of non-protein nitrogen in the blood which is not furnished by the simpler plan of estimating the blood-urea alone. The pregnancy should be terminated if the blood-urea rises above 40 mgms., whether the case is one of nephritic or pre-eclamptic toxæmia.

The physician's most difficult task comes when the woman has been delivered, his task of deciding whether the albuminuria bespeaks a permanently damaged kidney. If the albuminuria was pre-eclamptic in origin the prognosis for both mother and child in subsequent pregnancies is good. It is bad for both if nephritis exists. If all trace of albumen and casts disappear and the blood-pressure falls to normal by the second or third week after delivery the case may be classified as pre-eclampsia, not of course with absolute certainty, but still with sufficient probability to permit the risk of another pregnancy. If the albumin and casts persist at the end of a month the case is probably one of nephritis. Sometimes there is an interval of six months to a year, and then, though no pregnancy intervenes, signs of definite nephritis reappear. In all cases, however, the state of the arteries and the blood-pressure will give valuable information. The presence of any degree of arterio-sclerosis and raised systolic and, in particular, diastolic pressures may be taken as proof of permanent renal damage. The most difficult case of all is that in which all signs and symptoms of nephritis are absent until the woman again becomes pregnant, and then after a few months all the old symptoms reappear.

So we have three classes :—

(*a*) Those in which the toxæmia was eclamptic. These may safely run the risk of another pregnancy.

(*b*) Those in which chronic nephritis can definitely be shown to be present. These cases should not be permitted to run the risk of another pregnancy.

(*c*) Those in which all signs of nephritis remained in abeyance until another pregnancy supervened. In such cases it must be borne in mind that each successive pregnancy becomes more dangerous to the mother, that the progress of her nephritis will be advanced, and that the chances of the child surviving become progressively poorer. Therefore the woman should be warned of the serious danger of repeated pregnancies. Should she again become pregnant the advisability of performing Cæsarean section if the pregnancy continues until the child becomes viable must be discussed, and the possibility of undergoing prophylactic sterilization must be offered to her.

### REFERENCES.

[1] Weichardt, in Llewellys Barker's *Endocrinology and Metabolism*, iv. 835.

[2] Von Leyden, *ibid.*

[3] Whitridge Williams, *Obstetrics.* Fifth Edition, *passim.* New York, 1927.

[4] Diday, *Infantile Syphilis*, p. 113, New Sydenham Society, 1859.

[5] Stafford and Addis, *Quart. Jour. Med.*, 1924, xvii. 151.

[6] McLean and De Wesselow, *Quart. Jour. Med.*, 1918–19, xii. 347.

